# Investigation on Creep Deformation and Age Strengthening Behavior of 304 Stainless Steel under High Stress Levels

**DOI:** 10.3390/ma17030642

**Published:** 2024-01-28

**Authors:** Lihua Zhan, Hao Xie, Youliang Yang, Shuai Zhao, Zhilong Chang, Yunni Xia, Zeyu Zheng, Yujie Zhou

**Affiliations:** 1 Research Institute of Light Alloy, Central South University, Changsha 410083, China; yjs-cast@csu.edu.cn (L.Z.); lzhaoeds1112@163.com (S.Z.);; 2 State Key Laboratory of Precision Manufacturing for Extreme Service Performance, Central South University, Changsha 410083, China; 3 College of Mechanical and Electrical Engineering, Central South University, Changsha 410083, China

**Keywords:** creep deformation, 304 stainless steel, mechanical properties, dislocation density, constitutive model

## Abstract

The creep deformation behavior and age strengthening behavior of 304 stainless steel under high stress levels were systematically studied by uniaxial creep test, tensile test, XRD diffraction test and transmission electron microscopy. The results show that the total creep strain and the initial creep strain rate increase with the increase in stress level, and the creep strain in the whole aging process is mainly produced in the initial creep stage. The calculated stress exponent shows that the main mechanism of creep deformation of 304 stainless steel at 453 K is dislocation slip. The strength and plasticity of 304 stainless steel after creep aging are improved simultaneously. Microstructural observations indicate an increase in dislocation density and martensite content, as well as austenite and twins, leading to an improvement in strength and plasticity, respectively. In addition, considering the influence of dislocation density on creep behavior, the relative dislocation density increase is introduced into the hyperbolic sine creep model, and a simple mechanism-based creep aging constitutive model is established. The creep strain predicted by the model is in good agreement with the experimental data of 304 stainless steel. The findings can provide theoretical support for the application of creep age forming in 304 stainless steel parts.

## 1. Introduction

With the continuous development of new technologies, the composition of steel has gradually evolved from the initial two elements of C and Fe to a composite iron-based alloy with Fe as the main element and a large number of other alloying micro-elements added [1]. 304 stainless steel (304 SS) is widely used in aerospace, transport medical and other civil and military fields due to its good low-temperature performance, forming properties, welding performance and low cost [2,3,4,5]. For example, SpaceX’s rocket fuel tanks are made of 304 SS, which significantly improves productivity and reduces costs. 304 SS is a metastable austenitic stainless steel (MASS) where deformation below M_d_ temperature can induce transformation from the fcc austenite phase (*γ*) to the bcc martensite phase (*α*′) [6]. M_d_ temperature is a temperature above which deformation does not induce martensitic transformation. Various studies of MASS plastically deformed at room temperature have shown that dissociated dislocations, laminar dislocations, deformation twins and ε-martensite with an hcp structure occur within the austenite [7,8,9,10]. These microstructures, which can be collectively referred to as shear zones, tend to occur during plastic deformation of materials with low-stacking fault energy, and α’-martensite tends to nucleate and grow at the location of such shear zones and their intersections [6,7,8,9,10,11]. At the same time, 304 SS is also a transformation-related induced plasticity (TRIP) steel. The gradual transformation of the remaining austenite to martensite induces a significant work-hardening behavior, which enhances both the strength and plasticity of the material [12,13,14,15]. 

Creep age forming (CAF) is a kind of advanced sheet forming technology applicable to the manufacture of large and complex metal components, during which the component to be formed can simultaneously achieve the desirable configurations and enhanced performance through creep deformation and aging precipitation. It has attracted more and more attention in the aerospace sector due to its advantages of good performance and high precision of its formed components [16,17]. Yang et al. investigated the stress relaxation aging behavior and microstructural evolution of aluminum–copper alloys under different initial stresses [18]. Liu et al. investigated the use of engineered dislocations in aluminum alloys to achieve large creep formability and strength–conductivity synergy [19]. Li et al. established a unified constitutive model for asymmetric tension and compression creep-aging behavior of a naturally aged Al-Cu-Li alloy [20]. In summary, it can be seen that the current creep aging mechanism research is mainly focused on aluminum alloy materials; however, there are few investigations on the creep age characteristics of stainless steel.

Up to now, the studies on stainless steel creep have mainly focused on the precipitation phase transition of stainless steel at high temperatures, void nucleation and growth, creep damage and creep life prediction [21,22,23,24]. Zhang et al. investigated the creep life prediction method for 304 SS at high temperatures (550 °C, 593 °C and 650 °C) [21]. Brnic et al. investigated the creep resistance of 304 SS at the temperatures of 400 °C and 500 °C for a short period of time (1 h) [24]. Petit et al. studied the microstructure evolution during deformation of 304 SS [14]. Tsukada et al. studied the correlation between defect energy and ferromagnetic phase precipitation in creep fracture experiments of 304 SS at 873 K [25]. Based on the above references, the current research on the creep temperature of 304 SS is mainly focused on 300–650 °C, i.e., the service environment temperature, and the research on the creep stage is also mainly the creep damage stage [26,27,28,29,30], and there is no consideration of the use of creep for the forming and manufacturing of 304 SS components. CAF primarily utilizes the first and second stages of creep to form the components at low temperatures for a short period of time, and there is yet to be a report on the mechanism of the CAF of stainless steel [17]. Therefore, it is of great significance to systematically explore CAF’s feasibility for stainless steel components in the aerospace field.

In recent years, due to the increasing carrying capacity of aerospace vehicles and the need for aerodynamic profiles, the structures of metallic components have evolved towards high stiffeners and complex curvature. During the bending loading process, the internal stress levels along the thickness direction of the member are often within the elastic–plastic deformation range, which may exceed the yield strength of the material [31]. The existing research is mainly concerned with the creep behavior of materials under stress levels below the yield strength [32,33,34], but the creep deformation and microstructure evolution of materials under high stress are less studied. Therefore, it is extremely necessary to carry out research on creep aging behavior under high stress for the creep aging process of complex thin-walled structural components. Based on a comprehensive analysis of the aforementioned literature, this paper intends to systematically carry out research on the creep behavior and strength evolution law of 304 SS under high stress, and the research findings will contribute to promoting the application of CAF to 304 SS thin-walled components.

This paper aims to study the creep behavior and age strengthening response of 304 SS under high stress levels. The calculated stress exponent and the microstructure characterization reveal the corresponding deformation and strengthening mechanisms. Furthermore, based on the dislocation density, a simple mechanism-based creep aging constitutive model is established for the 304 SS. The material parameters are calibrated using experimental data and the model prediction values and experimental results are compared and discussed.

## 2. Experimental

### 2.1. Material

**Figure 1:**
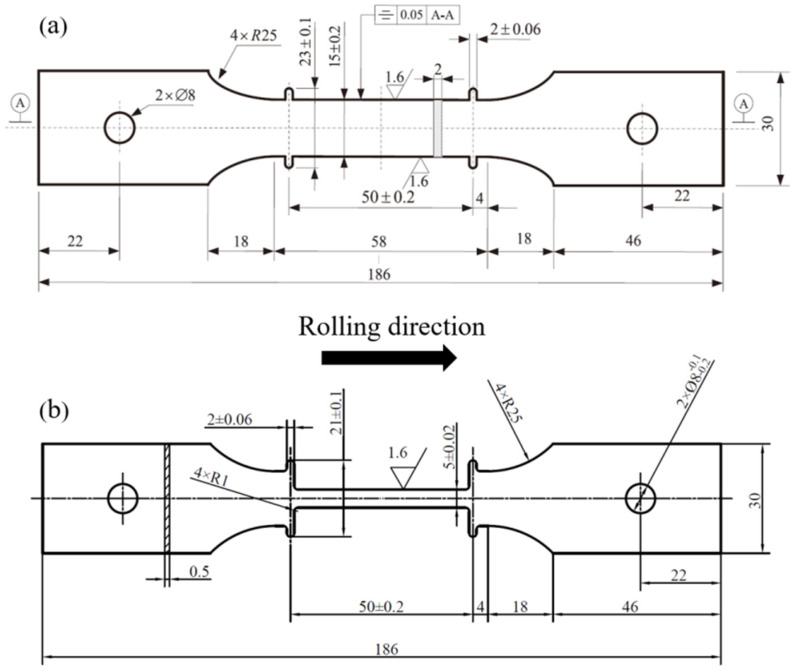
Specimen geometry (unit: mm): (**a**) standard specimen; (**b**) optimized specimen.

The raw material used in this study is a 304 SS hot rolled sheet of 0.5 mm thickness supplied by Tianjin Hangyu Zhuoran Technology Co., Ltd., Tianjin, China and its chemical composition is shown in Table 1. The chemical composition was measured by inductively coupled plasma optical emission spectroscopy (ICP-OES, SPECTRO BLUE SOP). The dimensions of the creep specimens according to ISO 204:2018 standards [35] are 186 mm total length, 50 mm gauge length, 15 mm gauge width and 2 mm thickness, as shown in Figure 1a. Due to the small thickness of the 304 SS raw material (0.5 mm), the width of the gauge area of the specimen was designed to be 5 mm to ensure a uniform strain, as shown in Figure 1b. Note that creep specimens were machined along the rolling direction of the plate. 

### 2.2. Experimental Procedure

The uniaxial tensile creep test was carried out on an RMT-D5 electronic high temperature creep tester manufactured by SUST. The creep specimen is first installed in the furnace of the creep tester, and then the extensomer is attached to the ridges at both ends of the gauge length to measure the strain and finally, a thermocouple thermometer is fixed in the gauge area of the sample. After finishing the sample installation, the equipment begins to heat up. When the thermocouple temperature reached the target value of 453 K and stabilized for 5 min, the sample began to be loaded to different stress levels of 360, 380 and 400 MPa for a holding time of 8 h. After the experiment, the sample was then unloaded to 150 N and cooled to room temperature. The error of loading and heating system are ±3 N and ±2 K, respectively. Note that the yield strength of 304 stainless steel at 453 K is about 300 MPa, which is lower than the stress levels in this study, and can accurately reflect the creep deformation behavior of complex thin-walled components after loading with large curvature.

Tensile testing of initial specimens and specimens after the creep aging test and artificially aged specimens was carried out at room temperature under a constant speed of 2 mm/min using a CMT-5105 tester manufactured by MTS. The tensile tests under each condition were repeated three times to obtain the average values. An X-ray diffractometer (XRD) was used to characterize the composition and relative content variations of some specific phases. 

The measured point plane spacing and diffraction intensities of the material were compared with the diffraction data of the standard phases to determine the phases present in the material. The diffractograms obtained were compared with those in the literature to determine the phase corresponding to each diffraction peak. The bulk samples with a size of 5 mm × 5 mm × 0.5 mm were cut from the raw materials and samples after the creep aging test by an electric spark wire cutting machine, and then the surfaces of the samples were mechanically abraded with 400#, 1000#, 1500# and 2000# water-abrasive sandpaper, respectively. Finally, in order to remove the strain layer produced by the sample due to friction during the mechanical grinding process, the grinding surface was electrolytically polished, and after the polishing was completed, the sample was rinsed with alcohol. XRD tests were carried out on a Smart Lab 3 kW X-ray diffractometer manufactured by Rigaku Co., Ltd., Tokyo, Japan with a scanning range of 30–100° and a scanning speed of 5°/min. In addition, the microstructures of the creep-aged and non-aged alloys were also observed using a Talos FEI 200X high-resolution transmission electron microscope (HRTEM) from Thermo Fisher Scientific, Waltham, MA, USA. Firstly, the TEM sample was mechanically thinned to 70–120 mm thickness and punched into a slice of 3 mm diameter, followed by double-jet electrolytic polishing in a solution containing 10% perchloric acid and 90% ethanol at a temperature of 0 °C and a voltage of 12 V.

## 3. Results and Discussion

### 3.1. Creep Deformation Behavior

**Figure 2:**
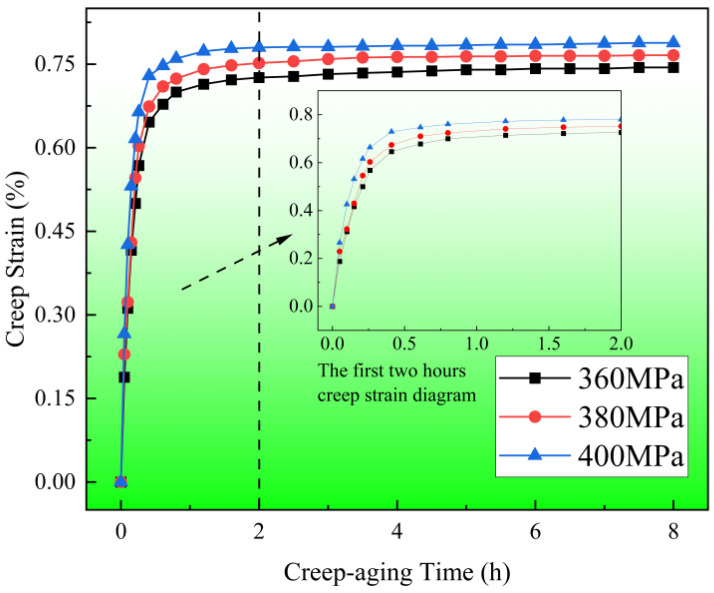
Creep strain–time curves of specimens at 453 K and different stress levels.

**Figure 3:**
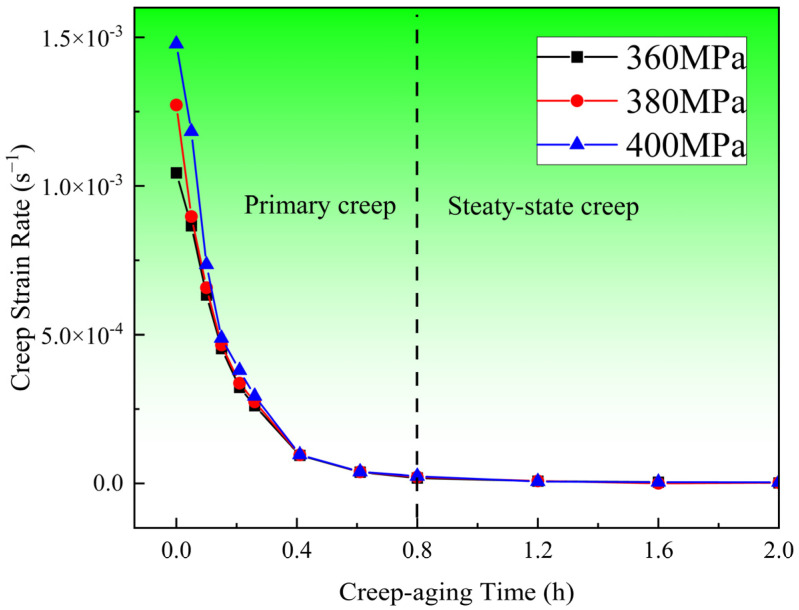
Creep strain rate–time curves of specimens at 453 K and different stress levels.

Figure 2 depicts the time-dependent creep strain curves of 304 SS at 453 K for 8 h with different stress levels of 360, 380 and 400 MPa. It can be seen that with the extension of time, all creep curves show a trend of rapid rise and then a slow increase. After creep aging for 8 h, the creep strain under three stress levels is relatively large, and the total strain is above 0.74%. The creep strain rate is obtained by differentiating the time of creep strain, as shown in Figure 3. Since the creep strain rate after creep aging for 2 h is basically unchanged and is very small compared with that before, only the first 2 h creep strain rate curves are displayed. As seen in Figure 2, the creep strain rate during the first 0.8 h of aging is significantly great and decreases rapidly, and after that, these curves enter into a steady-state stage with a constant strain rate.

**Figure 4:**
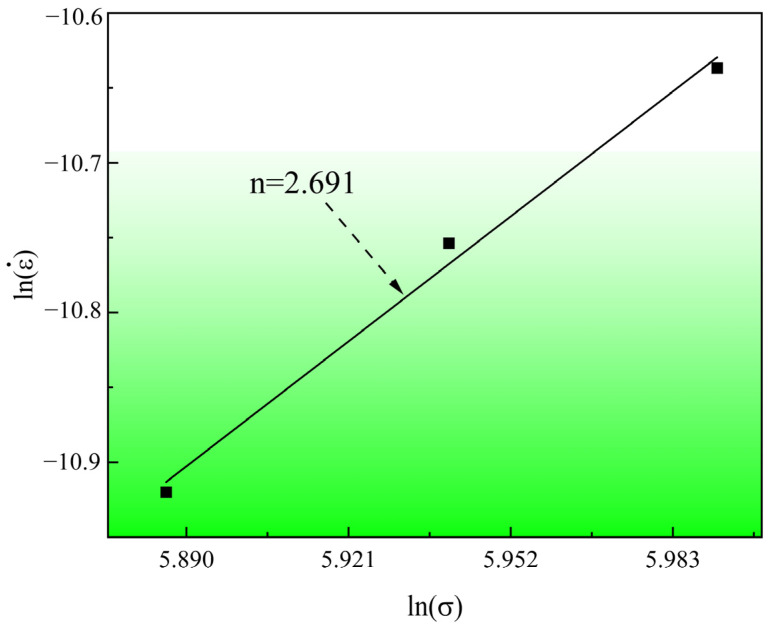
Log–log plot of steady creep strain rate-applied stress.

The relationship between steady creep rate, applied stress and aging temperature can be expressed by the following formula [35,36]:

(1) $$\overset{\cdot }{\varepsilon_{c}}=A\sigma^{n}\exp(-\frac{Q_{c}}{RT})$$

where $\dot{\varepsilon_{c}}$ represents the steady-state creep rate, A the material constant, σ the applied stress, $Q_{c}$ and R the apparent activation energy and gas constant (8.314 J/(mol·K)), respectively, T the thermodynamic temperature and n the stress exponent, which is obtained by fitting the slope of the logarithm of the steady-state creep rate and the logarithm of the stress. It is well-known that stress exponent can be used to reflect the creep mechanism [18,37,38,39]. Generally, n=1 indicates that the creep deformation is mainly controlled by the diffusion of vacancies in grains or along grain boundaries [40]; n=2~3 reveals that dislocation slip dominates creep deformation [41]. As shown in Figure 4, the stress exponent of 304 stainless steel creep aging at 453 K is 2.691 is between 2–3. It can be inferred that the mechanism of creep deformation is mainly dislocation slip. In the first stage of creep, with the increase of deformation, the total dislocation density increases, and the substructure is refined. In the second stage, when the creep reaches a steady state, the dislocation structure also reaches a steady state, and the dislocation structure does not change with time [42,43,44,45].

### 3.2. Mechanical Properties

Table 2 lists the yield strength, tensile strength and fracture elongation of 304 SS with different conditions. It can be seen from Table 2 that the yield strength of the material is slightly reduced after artificial aging, while the tensile strength and fracture elongation are relatively improved. Compared with the material after artificial aging, the yield strength of the material after creep aging is increased significantly, but the plasticity and tensile strength are decreased to a certain extent, and the greater the aging stress level, the more reduction in the plasticity. However, the yield strength, tensile strength and plasticity of the material after creep aging are greatly improved compared with the initial material. Specifically, the yield strength of the material decreases after artificial aging, from 463 MPa to 429 MPa, the tensile strength increases from 837 MPa to 1085 MPa and the elongation increases from 44.1% to 63.4%. This indicates that the material softens after artificial aging and its deformation resistance decreases; on the other hand, the work hardening ability becomes stronger, thus leading to the toughness and plasticity being improved. The difference between creep aging and artificial aging is whether there is stress applied to the specimen. Applied stress could make the material deform during aging. Because 304 SS is MASS, the material will produce a small amount of plastic deformation after loading. The dislocations in the material move along a slip plane under the action of stress. The dislocations will bend when encountering forest dislocations in the process of sliding or climbing, resulting in an increase in dislocation density and the formation of dislocation tangles or cellular structures. This dislocation structure is quite stable in mechanics and requires greater stress to move the dislocations again, thereby improving the yield strength of the material [46,47,48]. During tensile deformation, not only do deformation twins and dislocation proliferation occur, but the transformation of residual austenite to martensite is also induced, which simultaneously increases the plasticity and strength of the material, i.e., the TRIP effect [12,13,14]. Therefore, the plasticity and strength of the material after creep aging are improved compared with the initial material. The yield strength of the material after creep aging under different stress conditions is increased by 86, 94 and 98 MPa, respectively. The tensile strength is increased by 163, 220 and 278 MPa, and the elongation is increased by 13.1, 10.0 and 4.8%, respectively.

### 3.3. XRD Phase Analysis

**Figure 5:**
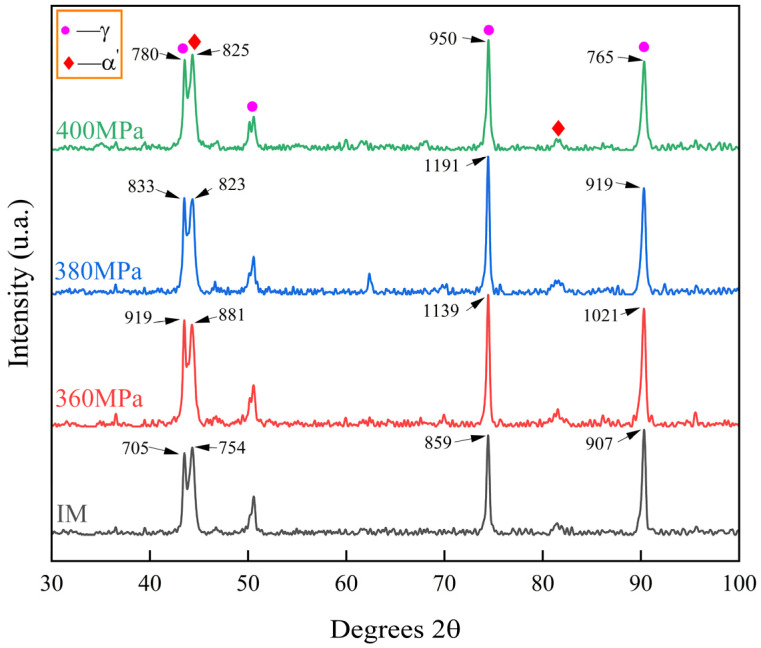
XRD patterns of 304 SS before and after creep aging under different conditions (the numbers at the arrows are the diffraction peak intensities).

The XRD diffraction patterns of the initial material (IM) and creep-aged samples are shown in Figure 5. The phase corresponding to each diffraction peak was determined by comparing the experimentally obtained XRD patterns with the XRD patterns in the literature [49,50,51]. From the diffraction diagram, it can be seen that most of the IM materials without creep aging are γ-austenite phase, with a certain amount of $\alpha^{\prime }$-martensite phase. According to the creep aging strengthening mechanism mentioned in the second section, as the creep aging stress level gradually increases, the creep strain also increases, that is, the strengthening effect also increases. From the strength of the diffraction peak (Figure 5), the strength of the diffraction peak of the martensite phase gradually increases after creep aging, indicating that the martensite content in the sample material is increasing. However, it can also be seen that the austenite diffraction peak intensity of the material after creep aging is higher than that of IM, which indicates that the austenite content of the material increases after creep aging. Additionally, the austenite content of 304 SS decreases as the stress level increases. This is because creep aging can provide a phase change driving force for the transformation of martensite, and during the loading process, the material undergoes plastic deformation, and the deformation increases crystal defects such as dislocations, stacking faults and twins. These defects provide favorable nucleation sites for the transformation of the martensite phase and also promote the nucleation and growth of the martensite phase from the austenite phase, and thus the austenite phase content is reduced slightly with the stress level [2,10,51]. In summary, the austenite content and martensite content of the material increase after creep aging, which can also provide a basis for the simultaneous increase in plasticity and strength of the material after creep aging.

### 3.4. Dislocation Density Evolution

The XRD pattern was analyzed by JADE 6 software to obtain the full width at half maximum (FWHM), and then the dislocation density (ρ) was calculated as the initial condition of the modified Williamson–Hall formula [52,53,54]. The ρ could be obtained by fitting the following function describing its relationship with the broadening diffraction peaks [54]:

(2) $$∆K=\left(\frac{0.9}{D}\right)+\left(\frac{\pi Mb^{2}}{2}\right)\rho^{1/2}K^{2}{\bar{C}}_{hkl}$$

where ${\bar{C}}_{hkl}$ is the average contrast factor for each specific plane *hkl*, D is the crystallite size, M is the Wilkens arrangement parameter, b is the burgers vector and ρ is the dislocation density. High-resolution 2*θ* scans were performed to measure the effect of increasing creep stress levels on the broadening of the diffraction peaks. A pseudo-Voigt function was subsequently fitted to each diffraction peak in order to calculate the full widths at half-maximum (FWHM). Δ*K* is the broadened FWHM in reciprocal space given by:

(3) $$∆K=\frac{2\cos\theta \cdot ∆\theta }{\lambda }$$

where θ is the diffraction angle, λ is the wavelength of the X-ray and *K* is the diffraction vector defined by:

(4) $$K=\frac{2\sin\theta }{\lambda }$$

The differences in the average contrast factors (${\bar{C}}_{hkl}$) for different diffraction peaks are used to account for broadening anisotropy [52,54]:

(5) $${\bar{C}}_{hkl}={\bar{C}}_{h00}\left(1-q\left(\frac{h^{2}k^{2}+h^{2}l^{2}+k^{2}l^{2}}{h^{2}+k^{2}+l^{2}}\right)\right)$$

where ${\bar{C}}_{h00}$ and q represent different broadening anisotropies and vary depending on whether dislocations are edge or screw in character.

**Figure 6:**
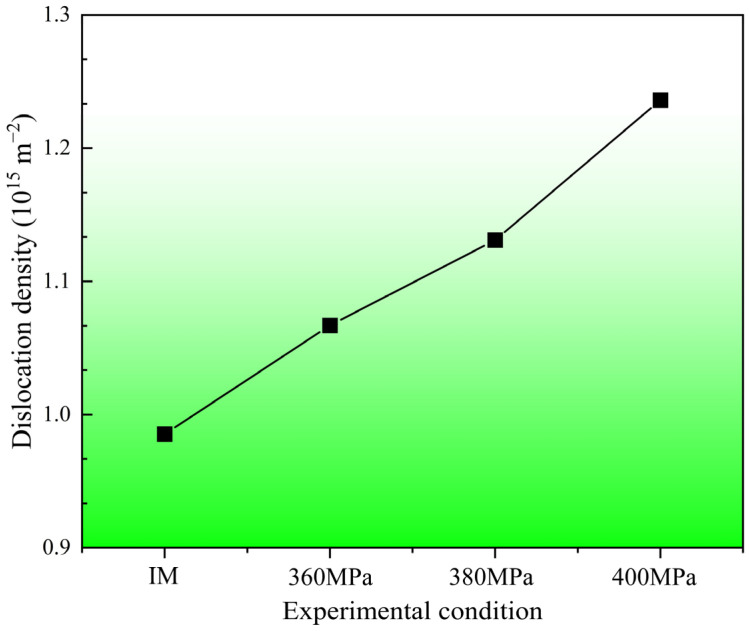
Dislocation density of 304 stainless steel before and after creep aging.

The dislocation density of the initial material (IM) and the samples after creep aging under different stress conditions is shown in Figure 6. It can be seen that the dislocation density of the material before creep has reached 9.853 × 10^14^ m^−2^. After creep aging, the dislocation density of the material increases linearly with the increase in stress level. During the creep aging process, the deformation mechanism of 304 SS is principally dominated by the dislocation slip. With the increase of deformation, the slip of dislocations would be hindered by the phase boundary and grain boundary, resulting in a serious pile-up and thus making them more difficult to deform. When the slip of dislocation is difficult, the deformation can often be coordinated by twinning [55]. At this time, the slip continues with the help of twinning, and the twinning deformation process is relatively fast. The shear stress required for twinning deformation is greater than the shear stress required for slip [9]. When the orientation of the crystal is not conducive to slip, twinning deformation occurs. However, after a certain twinning deformation, the orientation of the crystal changes, so that some slip systems are in a favorable orientation and slip deformation can be carried out. Therefore, when the slip is difficult, the orientation relationship is changed by twinning to make the slip easier [56,57]. However, the twinning deformation is an auxiliary deformation and the resultant deformation is small; the main deformation mechanism is still dislocation slip.

### 3.5. Microstructure Characteristics

**Figure 7:**
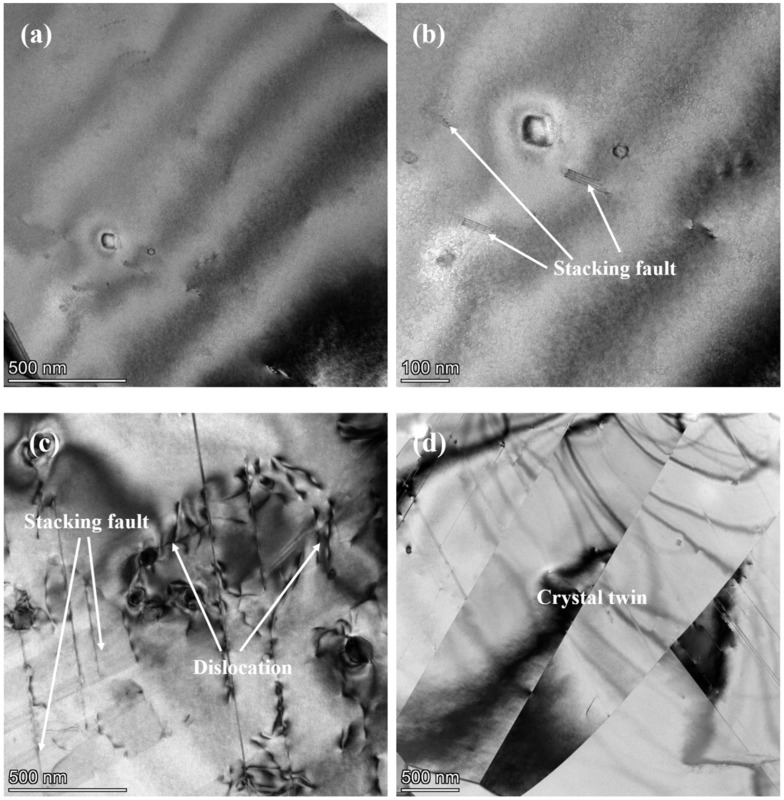
TEM images of the initial sample before creep aging: (**a**) internal microstructure characteristics of most grains; (**b**) stacking fault at high resolution; (**c**,**d**) defects in a few grains.

Figure 7 shows the microstructure of the initial material of 304 SS. As seen in Figure 7, a few dislocations are found inside the grain and the degree of lattice distortion is small. There are also stacking faults sporadically distributed within a small number of grains (Figure 7b,c). In addition, twins are observed in the matrix (Figure 7d). This may be due to the fact that the stacking fault energy of 304 SS is low, thus resulting in the generation of deformation twins during the grinding process of the sample [55]. Additionally, the straight twin boundaries in the grains are clearly visible. The existence of deformation twins will slightly increase the deformation degree of the material, and consequently, the mechanical properties of the material show the improvement in the plasticity of the material.

**Figure 8:**
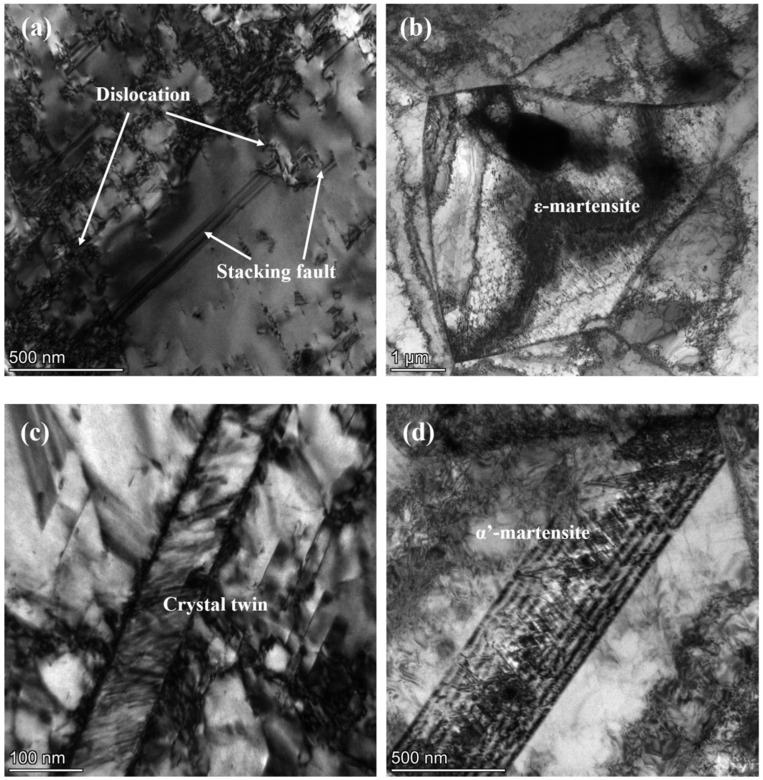
TEM images of samples after creep aging at 453 K–400 MPa–8 h: (**a**,**c**) defects in most grains; (**b**,**d**) martensite of different forms.

Figure 8 displays the microstructure of the 453 K–400 MPa–8 h creep aging sample. A large number of high-density dislocations are formed in the austenite (Figure 8a), which strengthens the austenite phase in the parent material and produces a mechanically stabilized phenomenon. And dislocations are also accumulated at the grain boundaries. At the same time, as the creep strain continues to increase, there will be part of the austenite transformation to ε-martensite, and then to $\alpha^{\prime }$-martensite transformation, or directly into $\alpha^{\prime }$-martensite [6], resulting in a rapid increase in the content of $\alpha^{\prime }$-martensite. It can be seen that $\alpha^{\prime }$-martensite inserts across the austenite crystal (Figure 8d), and these locations are the dense areas of dislocation rings or the areas where twins exist (Figure 8c). Moreover, the twinned crystals can also bend at the grain boundaries due to increased strain, resulting in the presence of untransformed ε-martensite at the shear zone and the presence of ε-martensite at defects in the austenite (Figure 8b) [6,9,12]. It can be inferred that the main deformation mechanism during creep aging is dislocation slip. After creep aging, stacking faults and twin crystals are generated and accompanied by martensitic transformation, which is consistent with the previous XRD experimental results. Therefore, the strengthening mechanism of creep aging was further revealed; that is, the increase in austenite content and the creation of deformation twins improve the plasticity of the material, while dislocation proliferation as well as the increase in martensitic phase content elevates the strength of the material. The microstructural changes obtained experimentally are consistent with the deformation and strengthening mechanisms analyzed before. 

## 4. Constitutive Modeling

### 4.1. Dislocation Density

During metal hot working deformation, there are always two processes of work hardening and dynamic recovery softening [58]. The dislocation density increases during the work hardening process and decreases during the dynamic recovery softening process. The change of dislocation density depends on the result of the competition between these two processes. According to Reference [59], the change of dislocation density in the process of work hardening and dynamic recovery satisfies:

(6) $$\frac{d\rho }{d\varepsilon }=h-r\rho_{0}$$

where $\rho_{0}$ is the initial dislocation density; $\frac{d\rho }{d\varepsilon }$ is the increase of dislocation density with the increase of strain; h represents the proliferation effect of work hardening on dislocation density; r is the dynamic recovery coefficient, which characterizes the annihilation of dislocation density by dynamic softening. ε is strain. Integrating Equation (7) yields:

(7) $$\rho =\rho_{0}e^{-r\varepsilon }+\frac{h}{r}(1-e^{-r\varepsilon })$$

Here, the concept of relative increase of dislocation density is introduced, as shown in Equation (8) [32]:

(8) $${\bar{\rho }}_{RI}=\frac{\rho {|}_{\sigma =\sigma_{i}}-\rho_{0}}{\rho_{0}}$$

Substituting Equation (8) into Equation (7), the relationship between the final relative dislocation density increases and the strain is as follows:

(9) $${\bar{\rho }}_{RI}=(1-e^{-r\varepsilon })(\frac{h}{r\rho_{0}}-1)$$

**Figure 9:**
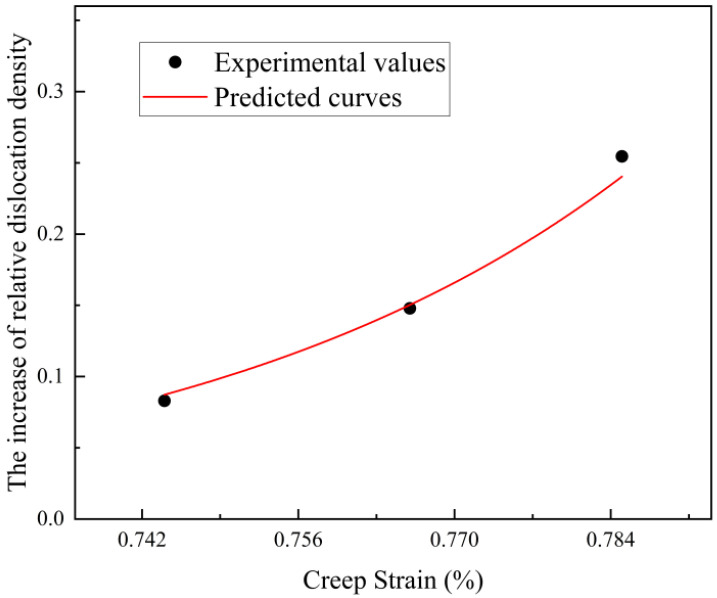
Relative dislocation density increase–creep strain fitting curve.

The dislocation density data and creep strain data measured by XRD diffraction experiment are used as input data to be fitted by Equation (9). The value of the material constant in the equation is: r=−24.71 ;h=−2.50, and the fitting curve of the relative dislocation density increases, and the creep strain is obtained, as shown in Figure 9. It can be seen from the figure that the increase of relative dislocation density increases with strain, and the larger the strain, the greater the increase rate of relative dislocation density.

### 4.2. Creep Aging Constitutive Model

According to the continuum damage mechanics model established by Kowalewski, the model describes the three stages of the entire creep [60]. In this study, the hyperbolic sine model is used to describe the creep behavior of 304 austenitic stainless steel. Because the material does not enter the third stage of creep, Huang et al. [61] ignored the damage state variables when modeling the creep age forming process. Then they proposed a constitutive equation for creep age forming after some simplifications and modification, as shown in Equation (10). The model has also been used by many scholars to simulate the creep aging behavior of materials [62].

(10) $$\left\{ \begin{array}{l} {\dot{\varepsilon }}_{c}=A\sinh[B(\sigma -\sigma_{0}){\left(1-H\right)}^{m_{0}}]\\ \dot{H}=\frac{h^{\prime }}{\sigma^{n}}\left(1-\frac{H}{H^{*}}\right){\dot{\varepsilon }}_{c} \end{array}\right.$$

where $A,\;B,{\;m}_{0},\;n,\;H^{*}$ are the material constants, which can be obtained by numerical fitting; $\sigma_{0}$ is the threshold stress; H represents the effect of work hardening on the initial creep behavior; $h^{\prime }$ is related to the work hardening effect.

In order to describe the creep behavior of 304 SS more accurately, considering that the main mechanism of creep deformation is dislocation slip and dislocation density has a certain influence on creep deformation, the increase of relative dislocation density is introduced into Equation (10), and then the creep aging constitutive modeling is established as follows:

(11) $$\left\{ \begin{array}{l} {\dot{\varepsilon }}_{c}=A\sinh[B(\sigma -\sigma_{0}){\left(1-H\right)}^{m_{0}}{\left(1+{\bar{\rho }}_{RI}\right)}^{m_{1}}]\\ \dot{H}=\frac{h^{\prime }}{\sigma^{n}}\left(1-\frac{H}{H^{*}}\right){\dot{\varepsilon }}_{c} \end{array}\right.$$

**Figure 10:**
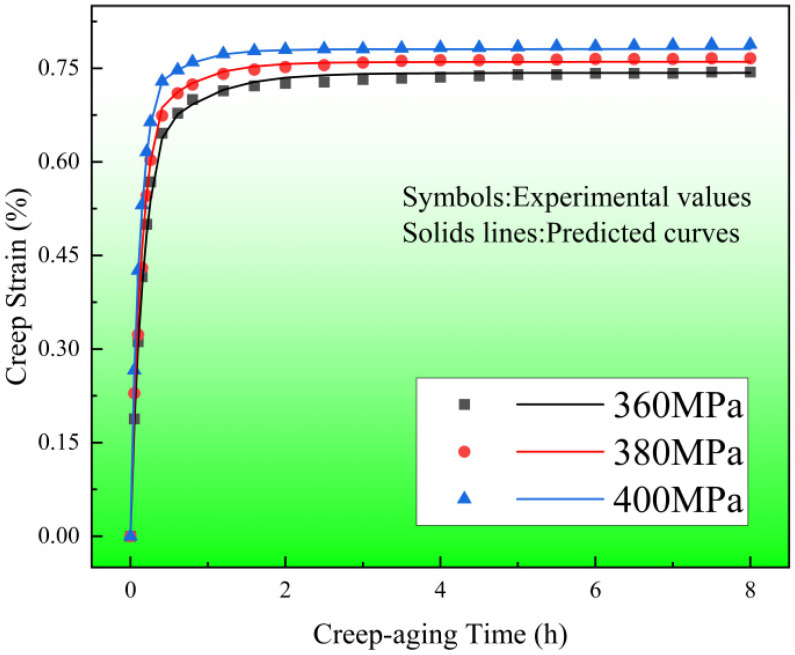
Comparison of creep strain predicted values and experimental values under different stress levels.

The particle swarm optimization algorithm is used to fit and optimize the constitutive modeling and the material constants of the creep aging constitutive modeling of 304 SS are obtained as shown in Table 3. The predicted creep strain values of 304 SS under different stress levels were compared with the experimental values, as shown in Figure 10. The results show that the creep curves fitted by the constitutive model are in good agreement with the actual creep curves. This proves that the established model can well describe the creep aging behavior of 304 SS under different high stresses.

## 5. Conclusions

The creep deformation and mechanical properties of 304 SS under high stress levels were studied experimentally. The deformation and strengthening mechanism of 304 SS was revealed through a series of experiments at different stress levels. A creep aging modeling of 304 SS was developed to characterize the creep aging behavior at different stress levels. The key conclusions drawn from the study are as follows:

(1) The creep aging behavior of 304 SS in the experiment is divided into two stages: the initial creep stage and the steady creep stage. The creep strain is mainly generated in the initial creep stage, and the creep strain rate in the first 0.8 h aging is significantly higher than that of the steady-state creep stage. The creep strain and the initial creep strain rate increase with the increase in stress level.
(2) The stress exponent shows that the main mechanism of creep deformation is dislocation slip within the stress range studied, and creep aging is accompanied by defect generation and phase transformation, thus strengthening the material. Moreover, the increase in the austenite content and deformation twins’ formation during creep aging collectively improve the plasticity of the material. Thus, both the strength and plasticity of the material elevate after creep aging.
(3) A simple mechanism-based creep aging constitutive modeling is established by introducing the increase of relative dislocation density. The material parameters in the modeling were determined using experimental data. The creep strain predicted by the modeling is in good agreement with the experimental results of 304 SS.

## Figures and Tables

**Table 1:** Chemical composition of 304 SS (wt. %).

C	Si	Cr	Mn	Ni	P	S	Fe
0.050	0.530	18.860	1.650	8.250	0.040	0.003	Bal.

**Table 2:** Mechanical properties of 304 SS before and after creep aging.

Experimental Condition	Yield Strength/MPa	Tensile Strength/MPa	Elongation/%
Before creep (initial material)	463	836.9	44.1
453 K–0 MPa–8 h (artificial aging)	429	1084.8	63.4
453 K–360 MPa–8 h	549	999.9	57.2
453 K–380 MPa–8 h	557	1057.0	54.1
453 K–400 MPa–8 h	561	1114.8	48.9

**Table 3:** The value of material constants of 304 SS.

Parameter	Value	Parameter	Value
A	0.10863	$m_{1}$	8.5512 × 10^−4^
B	0.012193	$h^{\prime }$	0.73109
$\sigma_{0}$	284.25	$H^{*}$	2.0361
$m_{0}$	0.22343	n	0.83710

## Data Availability

The processed data required to reproduce these findings cannot be shared at this time as these data form part of an ongoing study.
